# Sabretoothed Carnivores and the Killing of Large Prey

**DOI:** 10.1371/journal.pone.0024971

**Published:** 2011-10-19

**Authors:** Ki Andersson, David Norman, Lars Werdelin

**Affiliations:** 1 Department of Earth Sciences, University of Cambridge, Cambridge, United Kingdom; 2 Department of Palaeozoology, Swedish Museum of Natural History, Stockholm, Sweden; University of Maryland, United States of America

## Abstract

Sabre-like canines clearly have the potential to inflict grievous wounds leading to massive blood loss and rapid death. Hypotheses concerning sabretooth killing modes include attack to soft parts such as the belly or throat, where biting deep is essential to generate strikes reaching major blood vessels. Sabretoothed carnivorans are widely interpreted as hunters of larger and more powerful prey than that of their present-day nonsabretoothed relatives. However, the precise functional advantage of the sabretooth bite, particularly in relation to prey size, is unknown. Here, we present a new point-to-point bite model and show that, for sabretooths, depth of the killing bite decreases dramatically with increasing prey size. The extended gape of sabretooths only results in considerable increase in bite depth when biting into prey with a radius of less than ∼10 cm. For sabretooths, this size-reversed functional advantage suggests predation on species within a similar size range to those attacked by present-day carnivorans, rather than “megaherbivores” as previously believed. The development of the sabretooth condition appears to represent a shift in function and killing behaviour, rather than one in predator-prey relations. Furthermore, our results demonstrate how sabretoothed carnivorans are likely to have evolved along a functionally continuous trajectory: beginning as an extension of a jaw-powered killing bite, as adopted by present-day pantherine cats, followed by neck-powered biting and thereafter shifting to neck-powered shear-biting. We anticipate this new insight to be a starting point for detailed study of the evolution of pathways that encompass extreme specialisation, for example, understanding how neck-powered biting shifts into shear-biting and its significance for predator-prey interactions. We also expect that our model for point-to-point biting and bite depth estimations will yield new insights into the behaviours of a broad range of extinct predators including therocephalians (gorgonopsian + cynodont, sabretoothed mammal-like reptiles), sauropterygians (marine reptiles) and theropod dinosaurs.

## Introduction

The repeated evolution of spectacularly enlarged canines in Tertiary carnivorans [1] is often attributed to a major shift in preference for predation on very large-bodied forms, such as elephants, rhinos and other contemporary ‘megaherbivores’ [1], [2], [3], [4], [5]. Intuition suggests a straightforward relationship between jaw size and prey size and attempts to understand and explain the predatory habits of sabretoothed carnivorans have focused on the biomechanics of the sabretooth jaw systems. Previous work has showed that, coupled with the evolution of sabre-like canines was the shift from jaw-powered killing bite, as adopted by present-day pantherine cats [6] to neck-powered biting [7] with a centre of rotation (a ‘virtual hinge) located somewhere behind the head [8], a point around which muscles recruited from the neck region drove the bite in a head nodding-fashion [9]. With this reorganisation of the jaw system, i.e. the shift in position of the pivot point for the cranium to the back of the neck, the jaw now gains a virtual portion extending beyond the physical cranio-mandibular joint, which results in an increased effective size of the gape and bite, without physically increasing the length and size of the jaw. Previous work has also examined sabretooth attack and killing behaviour and a number of conflicting killing models have been suggested; these have involved stabbing, aided by neck-flexing [10], dynamic-stabbing [3], slicing [2] and shear-biting [11]. The exact location of the virtual hinge has been debated for nearly a century. Early stabbing models placed the virtual hinge in the caudalmost cervical region [3], [10]. In contrast, detailed examination of the anatomy of the neck [7] and mastoid region [9] suggested a virtual hinge located close to the skull near the atlanto-occipital joint, as predicted by the shear-biting model [11]. Gape and biting are also well understood in terms of muscular action and bite force [12], [13], [14], [15].

Although sabretooth skulls and jaw-systems are well understood in terms of morphology and mechanics, the precise interaction between predator and prey remains unexplained. No doubt predators with large elongated canines and bigger gapes are capable of delivering bigger bites, but what happens when prey becomes considerably larger than the predator? How does biting scale with increasing size of prey and predator and what bite depths are generated? For a predator, ability to reach critical structures set deep inside the body such as the belly [11] or throat [7] dramatically affect the potency of the killing bite [16], [17], [18], thereby reducing the risk of injury to the predator during the kill as the time of predator-prey interaction is minimised ([8], [19], [20]). To address this we modelled the principal factors associated with point-to-point biting and examined a range of present-day non-sabretoothed and fossil sabretoothed carnivorans.

## Results and Discussion

### Point-to-Point Biting

**Figure 1:**
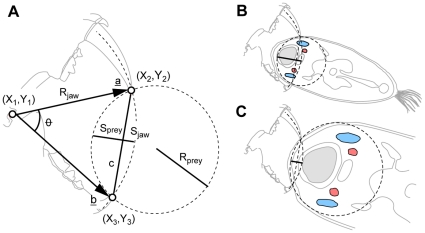
The outline of the prey modelled as a circle. In the canine clearance model bite is restricted by what can be fitted between the tips of the canines at maximum jaw extension. (a) Basic circle geometry determines the depth of the bite (*h = S_prey_+S_jaw_*) into a prey of radius (*R_prey_*) for canine clearance (*c*) and jaw size (*R_jaw_*). (b) Illustrating the geometry of biting into prey of different sizes - a sabretooth may deliver a fatal wound when biting the neck of the prey. (c) At twice the prey size the same sabretooth is capable of delivering a superficial bite only. Showing veins (blue), arteries (red), trachea (grey).

The model for point-to-point killing bites presented here brings together two aspects, first the relationship between canine size and gape, and second, between prey size and bite depth, the latter ultimately the main factor behind the killing potency of the bite. The first and fundamental assumption of point-to-point biting is that there is a relationship between the size of the canines and the amount of clearance between the tips of the canines at maximum gape. This relationship, here referred to as “canine clearance” is optimised when the combined height of the upper and lower canines equals the amount of clearance between the tips of canines at maximum gape. Secondly, circular geometry closely approximates a strike into a curved outline of a prey animal (Fig. 1), and hence maximum theoretical bite depth is determined as the interaction of two circles, one representing the jaw of the predator with radius *R_jaw_* and the other representing the prey with radius *R_prey_* (Fig. 1a). In figure 1 the neck was chosen to illustrate prey radius. The model however is not restricted to neck-bites only nor does it assume it, on the contrary, it may equally well apply to any curved part of the body.

**Figure 2:**
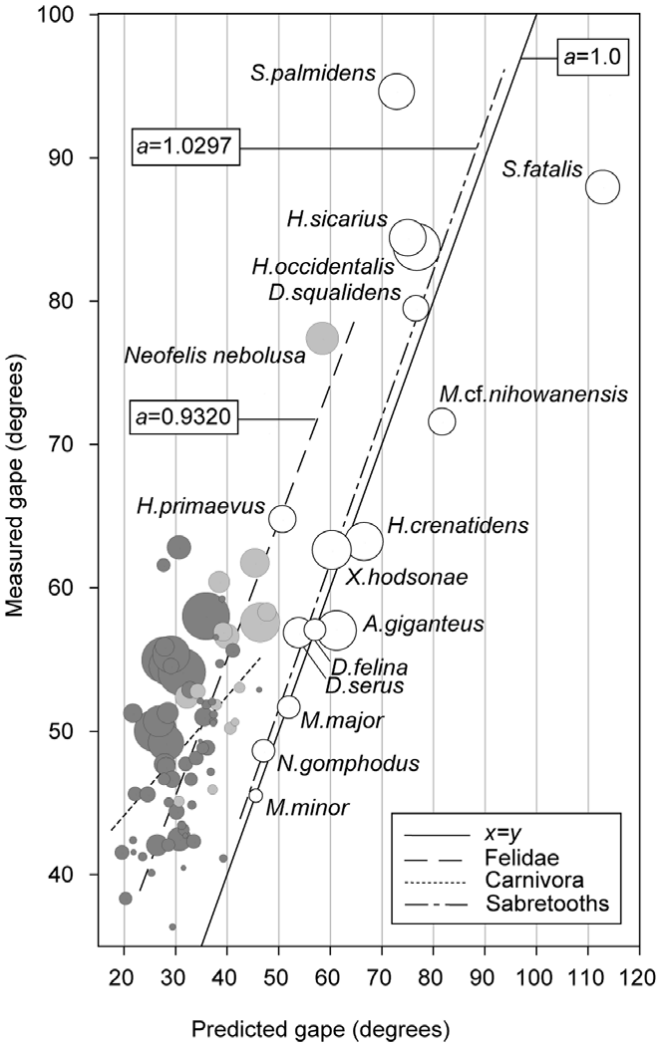
Canine size follows gape for carnivorans with canine killing bite habits. Measured gape plotted against gape predicted from canine size, for fossil sabretooths (white symbols and dash-dot regression line), present day non-sabretoothed carnivorans (Felidae, light gray symbols and dashed regression line; all other carnivoran families, dark grey symbols and dotted regression line). The solid line marks isometry (*y* = x) between measured and predicted gape and bubble diameters represent *R_shift_* values (not to scale). Gape is measured as the angle formed between the craniomandibular-joint and the tips of the incisors. Gape is predicted assuming canine clearance equal to the combined height of the upper and lower canine and calculated as the sum thereof.

To test canine clearance and its assumptions for point-to-point biting we compared measured actual gape angle at maximum jaw extension to gape angles predicted for an optimal canine-height and gape configuration for a range of extant and extinct carnivorans (Fig. 2). Gape angles were predicted by assuming a one to one relationship between the combined crown height of the upper and lower canines and the distance or “clearance” between the tips at maximum jaw extension. For canids, which typically kill large prey with multiple bites [21], measured- and predicted gape were loosely correlated (Linear Regression (LR); *y*0 = 16.838, *a* = 1.141, R^2^ = 0.701, SEE = 2.973, P = 0.0013, *n* = 11) and they were completely decoupled for Viverridae-Herpestidae (LR; y0 = 43.386, *a* = 0.064, R^2^ = 0.003, SEE = 5.519, P = 0.802, *n* = 36), all frugivorous, omnivorous or carnivorous hunters of small prey. For Ursids (*n* = 4) and for Hyaenids (*n* = 3) there was a very low fit between measured and predicted gape, reflecting the back-molar crushing employed by bears and in hyenas bone-cracking using third premolars.

For extant non-sabretoothed felid genera, measured gape and gape predicted by optimal canine clearance are significantly correlated (LR, *y*0 = 17.106, *a* = 0.932, R^2^ = 0.684, SEE = 4.533, P = 0.0001, *n* = 15). Despite having by far the widest gape among present day felids, the measured gape angle for the clouded leopard (*Neofelis nebulosa*) is smaller than predicted, and similar to that of extant non-sabretoothed felids (Fig. 2). For present day felids, on average measured gape is approximately 17 degrees less than predicted gape. This offset suggests an emphasis on fitting as much of the prey as possible inside the mouth between the canines over deep canine penetration, thus reflecting the habit of dispatching large prey with a single killing bite [21] often of a compressive nature. This killing bite mode is also similarly reflected in a range of cranial features [22]. Sabretooths closely fit the canine clearance model, with the exception of *Smilodon* and *Megantereon*, the sabres of which extend well beyond their ability to gape, and there was loose but significant correlation between measured and predicted gape (LR; *y*0 = 1.0229, *a* = 1.0297, R^2^ = 0.7143, SEE = 8.2748, P = 0.0001, *n* = 14; *Smilodon* outlier and excluded to assure normality).

Our analysis shows that, sabretoothed carnivorans are capable of exceptional gapes and with the exception *Smilodon* and *Megantereon*, optimisation of sabre size relative to gape suggests a strong functional emphasis on the canine killing bite. For Smilodontini (*i.e. Smilodon, Meganteron*) gape and canine clearance appear to be decoupled, thus suggesting an additional functional component in addition to point-to-point biting, such as *e.g.* the shear-bite (*sensu* Akersten 1985).

### Bite Depth

**Figure 3:**
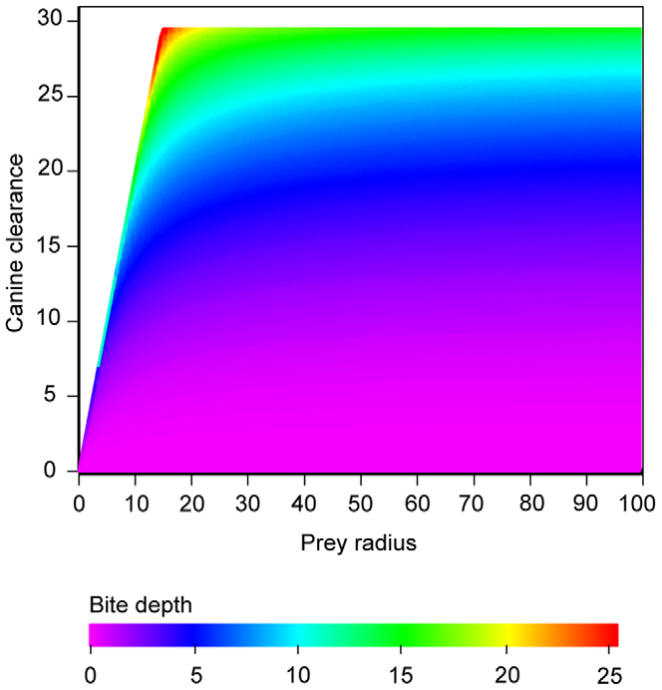
Bite depth rapidly drops with increasing prey radius. Contour plot of bite depth (*h*, z-axis) for a 15 cm jaw as a function of prey radius (*R_prey_*, x-axis) and canine clearance (*c*, y-axis) for two different jaw lengths. The arrow indicates how bite depth decreases as prey radius increases. The *R_shift_*-threshold around which small changes in canine clearance (*c*) shift from returning large to small bite depth to smaller than the change itself is shown as a straight line.

We modelled bite depth for predators and prey at various sizes, assuming optimal canine clearance. The results are presented in Fig. 3. The full implication of predator-prey scaling in point-to-point biting is illustrated by the following comparison. Consider a predator with a 15 cm jaw (*R*_jaw15_) and a 10 cm clearance (*c*_10_) between the canines at maximum gape, biting into prey with radii ranging between 1 and 100 cm (*R*_prey1…100_). Maximum theoretical bite depth is limited by what can be fitted between the canines and ranges between 10 and 5.86 cm. This can be achieved for prey with radius smaller than 5 cm. For prey with radius 5 cm (*R*_prey5_) bite depth is 5.86 cm and from there it drops dramatically to ∼2.2 cm for prey with a 10 cm radius (*R*_prey10_) and 0.98 cm for prey at 100 cm radius (*R*_prey100_). Now, consider the same jaw dimension but increase the gape and canines by 50%, from canine clearance 10 to 15 cm (*c*_15_). Maximum bite depth now ranges between 15 and 9.51 cm and is achieved for prey with less than 7.5 cm radius. At *R*_prey7.5_ bite depth is 9.51 cm, At *R*_prey10_ bite depth is 5.40 cm and at *R*_prey,100_ bite depth is ∼2.3 cm.

Increasing canine size and gape to a “sabretooth-like” condition has great impact on bite depth for small and medium sized prey but not for large prey. For a 10 cm radius prey a non-sabretoothed bite reaches 22% of the prey radius and an equally sized “sabretooth” reaches 54% of the prey radius. At 100 cm prey radius, however the same comparison is 0.98% and 2.30% of prey radius respectively, bites that can only be described as superficial.

**Figure 4:**
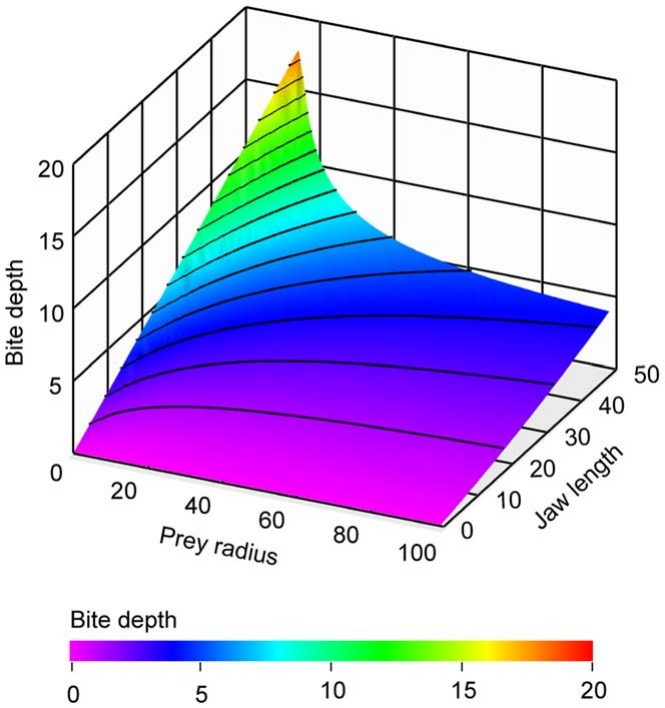
Bite depth increase with increasing jaw size. Bite depth (*h*, z-axis) plotted against jaw size (x-axis) and prey size (Y-axis) in 10 cm increments for a jaw with fixed canine clearance and jaw length proportion of 0.667 (i.e. 15 cm jaw length and 10 cm canine clearance).

By fixing the canine clearance to jaw size proportion the model can be used to predict how bite depth changes with increasing size. In figure 4 bite depth is presented for an 0.66 proportion (i.e.15 cm jaw and 10 cm canine clearance). For comparison the same value for the extant *Panthera* is 0.625. Throughout the size range larger predators deliver deeper bites than their smaller counterparts. The relationship between predator size and bite depth changes as prey become larger, however. For prey with 10 cm radius bite depth increases exponentially (y = 0.3704^1.1185x^, R^2^ = 0.989, P<0.0001) with increasing jaw size. For large prey (100 cm radius) the increase is close to linear, changing at a rate of 0.856 cm per 10 cm (y = −0.2458+0.0856x, R^2^ = 0.993, P<0.0001). Thus, in terms of bite depth, for the predator there is a relatively greater advantage in becoming larger when opting for small and medium sized prey than for large prey.

By increasing size or adopting the sabretooth condition, with elongated canines and extended gaping ability, a predator can deliver a substantially deeper bite to prey towards the smaller end of the prey spectrum. For large sized prey, or body-parts with a big radius, bites however remain superficial regardless of sabre-like canines or increase in size. Although determining the exact bite depth required to fatally injure or kill prey is beyond the scope of this study, biting to a depth of 50% of prey radius is clearly potentially more lethal than a couple centimetres into a prey of one meter radius. It should be added that carnivorans are known to reposition their bites during a kill and thus, where possible, to compress the prey and thereby increase bite depth. This prey compression is not included in the current model. Although this effect may be considerable it does not alter the geometric interaction between the jaw and the prey.

### Bite depth optimisation

**Figure 5:**
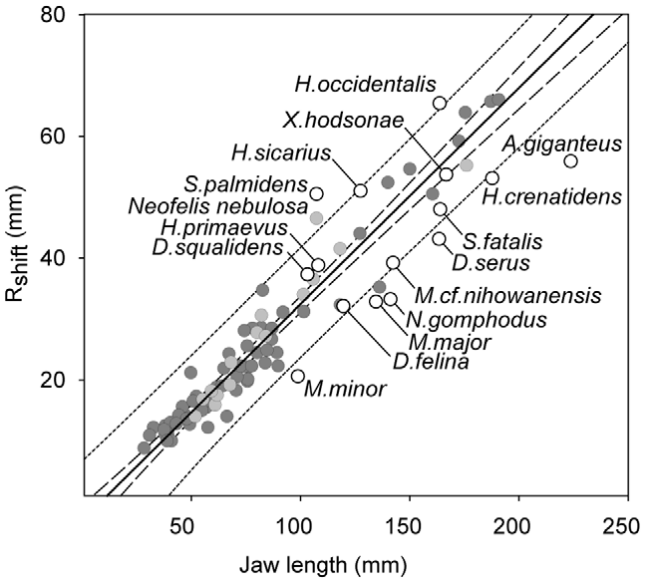
The prey size threshold around which bite depth changes. The prey radius (*R_shift_*) around which bite depth alters plotted against jaw length. Sabretooths, with the exception of *Amphimachairodus giganteus* has *R_shift_*_ values_ similar to extant felids of similar size. Extant Felidae, light grey circles; all other extant carnivoran families, dark grey circles; Sabretooth Felidae open circles and labelled. Linear regression (y^0^ = −3.103, a = 0.3553, R^2^ = 0.940, SEE = 3.4839, P = <0.0001) of all extant carnivoran families (sabretooths excluded). Confidence line (dashed) and prediction line (dotted) at 99%. Jaw length is the distance from the tip of the lower canine to the posterior end of the mandibular condyle.

Because bite depth change is differentiated over different-sized prey, we can determine the theoretical prey radius around which the resulting bite depth alters, here termed *R_shift_*. In other words bite depths for increasing prey radii up to the *R_shift_* threshold are relatively high and above the threshold the opposite applies (Fig. 5, see also supplementary information, Table S1, for *R_shift_*-values for each species analysed). For extant carnivorans the general trend is for *R_shift_* -values to increase at a rate of approximately 1 cm per every 3.6 cm jaw length (solid line in Fig. 5).

The low *R_shift_* values relative to size for ursids (*Ursus R_shift_* = 6.59 cm, *Melursus R_shift_* = 6.56 cm, *Selenarctos R_shift_* = 6.38 cm), hyaenids (*Crocuta R_shift_* = 5.91 cm, *Hyaena R_shift_* = 5.45 cm, *Proteles R_shift_* = 3.46 cm) and canids (*Lycaon R_shift_* = 5.23 cm, *Chrysocyon R_shift_* = 5.04 cm, *Canis R_shift_* = 4.39 cm) reflect functional emphasis on the post-canine dentition, also indicated by gape being greater than that predicted from canine size. On the other hand, high *R_shift_* values for extant non-sabretoothed pantherine cats (*Panthera leo R_shift_* = 6.53 cm; *P. tigris R_shift_* = 6.26 cm; *P. onca R_shift_* = 4.86 cm; *P. pardus R_shift_* = 4.47 cm), reflect the functional optimisation of the canine dentition and the habit of delivering single killing bites, as also indicated by the observation that measured gape closely follows predicted gape (Fig. 2). Values (*R_shift_*) also approximately match the size of prey and the structures commonly attacked, i.e. throats, necks, muzzles, etc. In relation to its very large size *Amphimachairodus giganteus* has a relatively low *R_shift_* (5.54 cm). Sabretoothed carnivorans do not have consistently higher *R_shift_* -values than pantherine cats, despite having wider gapes and larger canines.

### Implications for Sabretooth Bitemechanics and Evolution

Sabretoothed felids have been subdivided into scimitar-toothed cats [23], [24] (e.g. *Homotherium*) with characteristically short canines and long and slender limbs and the dirk-toothed cats [23], [24] (e.g. *Smilodon*) with long canines and short, powerful limbs. Such a clear division is not seen in our analysis, which rather adds support to the idea of a continuous functional spectrum as has been suggested previously [8], [24] whereby the evolution of sabretooth biting strategies progressed as a functional continuum, starting with the normal canine killing bite powered by the jaw adductor, *m. temporalis* and *m. masseter* [25] and followed by the neck-hinged bite, powered by the atlantomastoid *m. obliquus capitis cranialis* and *m. obliquus capitis caudalis* [7], [9]. In our analysis it is only necessary to infer the highly specialised sabretooth shear-bite (*sensu* Akersten 1985) killing model for *Smilodon* because of their large canine dentition, which does not match their canine clearance and gape and are thus not fully functional for biting. The existence of a functional continuum is supported by evidence from the clouded leopard (*Neofelis nebulosa*) with its intermediate sabre-/non-sabretooth morphology such as unusually large upper canines [26], [27] and from *Promegantereon ogygia* with its slender sabre-like canines and structurally intermediate, mastoid region [8] (*P. ogygia* was not included in the analysis because gape could not be determined on any known specimen). *Promegantereon* does not have the same level of canine specialisation as *Smilodon* and *Megantereon* thus making the tentative link between *Promegantereon* and Smilodontini [28] a transition of particular interest for the understanding of how extreme sabretooth specialisation evolves.

Although not directly recognised in this analysis, scimitar- and dirk-tooth adaptations may reflect hunting style rather than killing mode ([23]) and are not in conflict with the bite model presented here. On the contrary, viewing sabretooth development within the context of a functional continuum provides a novel framework against which to interpret taxa with apparently ‘puzzling’ mixes of anatomical features, such as *Xenosmilus hodsonae*, which has short canines combined with short massive limbs [29].

Although present-day carnivoran guilds are ecomorphologically diverse, no direct analogue of sabretooths exists today [30]. In modern ecosystems, energetic constraints determine when predators switch from small prey to prey as large as or larger than themselves [31]. Furthermore, present-day carnivorans generally avoid specialising on prey considerably larger than themselves [32], with the possible exception of the spotted hyaena (*Crocuta crocuta*) [33] and lions (*Panthera leo*) that readily prey upon species up to 3 times their own size [34]. Hunting in groups and being dominant members of the carnivoran guild also make lions less vulnerable to interspecific kleptoparasitism than a subordinate guild member [35]. Prey selection and killing mode are key to fully understanding the role of sabretooths in past carnivore guilds. The current analysis focuses on bite geometry and its constraints. Future work will have to look closer at the anatomy of potential prey and with the prey radii and bite depth constraints presented here in mind re-examine possible modes of killing. Questions such as to what parts of the body would a deep versus a shallow strike be fatal? Is multiple bite killing, with several bites directed at different parts or regions of the body an alternative to the single bite killing seen in most present day pantherine felids?

In summary, we have shown, that for multiple lineages of carnivoran sabretooths canine size and canine clearance are linked to gape, just as they are for present day felids. This suggests point-to-point biting and there is no need to invoke elaborate closed mouth, stabbing or slashing models to explain the function of sabre-like canines, except in the case of the uniquely specialised *Smilodon*.

We have also demonstrated why and how, contrary to popular perception, sabretooth jaws are not optimal for biting into large prey, and are in fact unsuited for this task. In combination with recent reviews of sabretooth skull morphology ([8], [19], [20]), neck anatomy [7], [9] and analysis of carnivoran palaeoguild structure [36], these results suggest strongly that sabretooths evolved for the fast and effective killing of prey within the same size range as those of their modern day non-sabretoothed relatives.

The insights presented here provide a functional and evolutionary framework for future studies on how changes to the remarkable sabretooth dentition are functionally, phylogenetically and developmentally linked to changes in skull architecture and modifications in the neck region and forequarters.

The model for point-to-point biting presented here is based on a fundamental bite geometric and is not carnivoran specific. Thus, it can be adopted to analyse a broad range of predators including therocephalians (gorgonopsian + cynodont, sabretoothed mammal-like reptiles [37]).

## Materials and Methods

### Model Assumptions and Data Collection

The model presented here brings together the following variables: maximum gape, canine size, jaw length, canine-clearance and prey radius to predict bite depth (Fig. 1). Maximum gape is the angle formed between the craniomandibular-joint and the tips of the incisors at maximum jaw extension, and canine height is crown height from which incisor height is subtracted. Jaw length is the distance between the tip of the lower canine and the mandibular condyle. Optimal canine clearance is the combined height of the upper and lower canines times two, assuming a for point-to-point biting optimal relationship between the size of the dentition and the amount that can be fitted between the tips at maximum jaw extension. When prey is modelled as a circle (Fig. 1), following basic plane geometry, canine clearance (*c*) is the chord of a line segment joining two points on a curve with radius (*R*) and bite depth (*h*) corresponds to the circle saggita. Bite depth is the combined interaction between one jaw-circle (jaw radius) and one opposing prey-circle (prey radius) and calculated as:
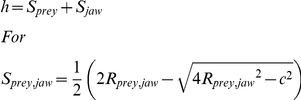
Bite depth (*h*) is the sum the jaw (*S_jaw_*) and the prey (*S_jaw_*) component for a jaw with a radius (*R_jaw_*) and canine-clearance (*c*) biting into a circular object with radius (*R_Prey_*). See fig. 1 for schematic illustration.

*R_shift_* is the point above which slope of *h* is >1 and below which it is <1. If prey radius (*R_prey_*) for canine clearence (*c*) is less than *R_shift_* then a small change in *R_prey_* causes a change in bite depth (*h*) equal or larger than the change itself. Calculated as the derivation of *R_prey_* on bite depth (*h*) with *S_jaw_* as a constant:
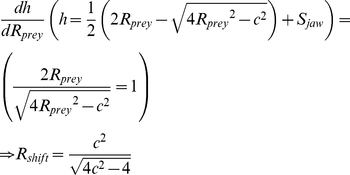
Data derives from Cartesian coordinates of 9 landmarks digitised from high-resolution digital images using tpsDig2 (http://life.bio.sunysb.edu/morph/index.html). Crown heights, gape angles and distances between canines, incisors and the craniomandibular-joint were determined using vector calculus and the following functions and variables:
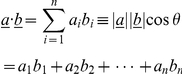


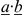
 is the scalar product of vectors of coordinates *a,b* and *θ* the gape determined as the angle between vectors.
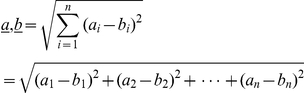

*a,b* is the vector magnitude determined as the Euclidean distance between coordinates *a,b*.

### Material

Data were collected from museum specimens and the literature. Altogether 269 individuals (155 species in 75 genera), representing the full range of present day carnivorans, and 16 specimens of 12 fossil sabretoothed genera were analysed.

The following sabretoothed felid and nimravid taxa were analysed: *Smilodon fatalis* LACMHC2001-2 [11], *Dinictis squalidens* AMNH 8777 [10], *Hoplophoneus primaveus* AMNH 11858 [10], *Sansanosmilus palmidens* Uncatalogued [10], *Nimravus gomphodus* AMNH 6933 [10]. *Dinictis felina* BC-603, *Dinobastis serus* TMM-933-3582, *Homotherium crenatidens* CB-06, *Hoplophoneus occidentalis* CB-18, *Hoplophoneus sicarius* CB-07, *Megantereon nihowanensis* BC-120, BC-20, *Metailurus major* PMU M3841, *Metailurus minor* PMU M3837, *Xenosmilus hodsonae* BIOPSI-101, *Amphimachairodus giganteus* MNCN uncatalogued cast.

Institutional abbreviations: LACMHC: Natural History Museum of Los Angeles County, Los Angeles, California. AMNH: American Museum of Natural History, New York. TMM: Texas Memorial Museum, Austin, Texas. PMU: Paleontological Section. Museum of Evolution, Uppsala, Sweden. BIOPSI: Babiarz Institute of Paleontological Studies, Inc., Mesa, Arizona. MNCN: Museo Nacional de Ciencas Naturales, Madrid, Spain. BC and CB are Bone Clones casting catalog numbers – original specimens from the collection of John P. Babiarz.

## Supporting Information

Table S1 Species and specimens included in the study. Canine clearance, measured gape, predicted gape, jaw-length and *R_shift_*-values for each species analysed.
(DOC)
