# Supplementary material for: Sabretoothed Carnivores and the Killing of Large Prey
Source: PLoS One. 2011 Oct 19;6(10):e24971. doi: 10.1371/journal.pone.0024971 (PMC3198467; doi:10.1371/journal.pone.0024971)
Supplement: Table S1 — Species and specimens included in the study. Canine clearance, measured gape, predicted gape, jaw-length and Rshift-values for each species analysed. (DOC) [file pone.0024971.s001.doc]

Supplementary information

Table S1

| Family | *Genus* | *Species* |  | n= species | n= speciemens | Canine clearance (mm) | Measured gape (deg) | Predicted gape (deg) | Jaw length (mm) | *R_shift_* (mm) |
| --- | --- | --- | --- | --- | --- | --- | --- | --- | --- | --- |
| Nandiniidae | *Nandinia* |  |  | 1 | 3 | 37.7 | 35 | 43 | 64.4 | 18.9 |
| Felidae | *Acinonyx* |  |  | 1 | 3 | 67.7 | 32 | 39 | 101.7 | 33.9 |
| Felidae | *Carcal* |  |  | 1 | 1 | 45.5 | 34 | 43 | 68.5 | 22.8 |
| Felidae | *Catopuma* |  |  | 1 | 1 | 55.5 | 39 | 47 | 80.4 | 27.7 |
| Felidae | *Felis* |  |  | 5 | 11 | 31.5 | 37 | 43 | 61.2 | 15.8 |
| Felidae | *Herpailurus* |  |  | 1 | 3 | 34.8 | 31 | 38 | 62.2 | 17.4 |
| Felidae | *Leopardus* |  |  | 2 | 6 | 38.2 | 41 | 48 | 68.0 | 19.1 |
| Felidae | *Lynx* |  |  | 2 | 4 | 54.2 | 48 | 55 | 84.4 | 27.1 |
| Felidae | *Neofelis* |  |  | 1 | 1 | 92.7 | 59 | 66 | 107.6 | 46.4 |
| Felidae | *Oncifelis* |  |  | 2 | 2 | 33.3 | 43 | 50 | 55.6 | 16.7 |
| Felidae | *Otocolobus* |  |  | 1 | 1 | 27.8 | 42 | 45 | 52.1 | 13.9 |
| Felidae | *Panthera* |  |  | 4 | 10 | 110.2 | 47 | 53 | 176.3 | 55.1 |
| Felidae | *Prionailurus* |  |  | 3 | 4 | 36.1 | 38 | 44 | 59.6 | 18.1 |
| Felidae | *Profelis* |  |  | 1 | 1 | 61.1 | 39 | 45 | 82.4 | 30.5 |
| Felidae | *Puma* |  |  | 1 | 3 | 72.8 | 40 | 49 | 106.1 | 36.4 |
| Felidae | *Uncia* |  |  | 1 | 1 | 82.8 | 46 | 52 | 118.5 | 41.4 |
| Viverridae | *Arctictis* |  |  | 1 | 1 | 45.3 | 36 | 38 | 84.1 | 22.7 |
| Viverridae | *Cynogale* |  |  | 1 | 1 | 56.8 | 22 | 28 | 78.3 | 28.4 |
| Viverridae | Genetta |  |  | 6 | 9 | 32.9 | 29 | 35 | 57.9 | 16.5 |
| Viverridae | *Hemigalus* |  |  | 2 | 2 | 37.4 | 28 | 34 | 61.9 | 18.7 |
| Viverridae | *Paradoxurus* |  |  | 2 | 2 | 56.0 | 28 | 34 | 74.5 | 28.0 |
| Viverridae | *Paguma* |  |  | 1 | 1 | 48.7 | 30 | 36 | 89.5 | 24.4 |
| Viverridae | *Viverra* |  |  | 3 | 4 | 49.6 | 30 | 36 | 85.0 | 24.8 |
| Viverridae | *Viverricula* |  |  | 1 | 1 | 24.3 | 30 | 36 | 57.9 | 12.1 |
| Hyaenidae | *Crocuta* |  |  | 1 | 3 | 118.1 | 26 | 34 | 172.8 | 59.1 |
| Hyaenidae | *Hyaena* |  |  | 2 | 3 | 109.1 | 29 | 36 | 150.3 | 54.5 |
| Hyaenidae | *Proteles* |  |  | 1 | 2 | 69.1 | 31 | 36 | 82.9 | 34.6 |
| Herpestidae | *Atilax* |  |  | 1 | 1 | 27.8 | 39 | 46 | 66.7 | 13.9 |
| Herpestidae | *Bdeogale* |  |  | 1 | 1 | 21.9 | 22 | 29 | 38.3 | 11.0 |
| Herpestidae | *Suricata* |  |  | 1 | 1 | 19.7 | 32 | 39 | 39.2 | 9.9 |
| Eupleridae | *Cryptoprocta* |  |  | 1 | 1 | 44.3 | 34 | 41 | 90.1 | 22.2 |
| Eupleridae | *Fossa* |  |  | 2 | 3 | 36.4 | 32 | 38 | 70.8 | 18.2 |
| Eupleridae | *Galidia* |  |  | 1 | 1 | 23.4 | 38 | 47 | 37.9 | 11.7 |
| Herpestidae | *Herpestes* |  |  | 5 | 5 | 29.7 | 31 | 40 | 55.2 | 14.9 |
| Herpestidae | *Ichneumia* |  |  | 1 | 1 | 48.4 | 29 | 34 | 67.6 | 24.2 |
| Herpestidae | *Mungo* |  |  | 1 | 1 | 19.9 | 32 | 39 | 41.2 | 9.9 |
| Canidae | *Alopex* |  |  | 1 | 3 | 61.9 | 29 | 35 | 92.3 | 31.0 |
| Canidae | *Canis* |  |  | 6 | 16 | 87.8 | 27 | 33 | 127.5 | 43.9 |
| Canidae | *Chrysocyon* |  |  | 1 | 2 | 100.7 | 28 | 33 | 160.7 | 50.4 |
| Canidae | *Cuon* |  |  | 1 | 3 | 64.3 | 27 | 34 | 118.5 | 32.2 |
| Canidae | *Dusicyon* |  |  | 5 | 9 | 62.1 | 28 | 34 | 101.8 | 31.1 |
| Canidae | *Lyacon* |  |  | 1 | 3 | 104.5 | 29 | 38 | 140.3 | 52.3 |
| Canidae | *Nyctereutes* |  |  | 1 | 1 | 48.8 | 25 | 31 | 80.5 | 24.4 |
| Canidae | *Otocyon* |  |  | 1 | 2 | 39.4 | 20 | 25 | 76.0 | 19.7 |
| Canidae | *Spetos* |  |  | 1 | 1 | 56.9 | 33 | 43 | 82.4 | 28.5 |
| Canidae | *Urocyon* |  |  | 1 | 1 | 44.1 | 20 | 25 | 76.6 | 22.0 |
| Canidae | *Vulpes* |  |  | 6 | 11 | 53.2 | 28 | 34 | 87.1 | 26.6 |
| Ursidae | Melursus |  |  | 1 | 2 | 131.1 | 36 | 39 | 187.4 | 65.6 |
| Ursidae | *Selenarctos* |  |  | 1 | 4 | 127.6 | 28 | 33 | 175.7 | 63.8 |
| Ursidae | *Tremarctus* |  |  | 1 | 3 | 70.2 | 31 | 38 | 136.5 | 35.1 |
| Ursidae | *Ursus* |  |  | 3 | 9 | 131.8 | 31 | 36 | 191.1 | 65.9 |
| Ailuridae | *Ailurus* |  |  | 1 | 1 | 44.4 | 22 | 27 | 72.8 | 22.2 |
| Mephitidae | *Mephitis* |  |  | 1 | 3 | 25.7 | 37 | 46 | 40.8 | 12.9 |
| Mephitidae | *Myadus* |  |  | 1 | 1 | 25.2 | 26 | 30 | 49.6 | 12.6 |
| Mephitidae | *Spilogale* |  |  | 1 | 2 | 17.4 | 35 | 44 | 28.7 | 8.7 |
| Mustelidae | *Aonyx* |  |  | 1 | 1 | 28.2 | 38 | 45 | 47.6 | 14.1 |
| Mustelidae | *Conepatus* |  |  | 1 | 1 | 30.9 | 39 | 47 | 46.3 | 15.5 |
| Mustelidae | *Eira* |  |  | 1 | 3 | 43.5 | 41 | 50 | 65.3 | 21.8 |
| Mustelidae | *Galictis* |  |  | 2 | 3 | 28.2 | 38 | 47 | 44.5 | 14.1 |
| Mustelidae | *Melogale* |  |  | 3 | 3 | 25.4 | 22 | 28 | 43.5 | 12.7 |
| Mustelidae | *Gulo* |  |  | 1 | 4 | 56.7 | 36 | 46 | 87.2 | 28.4 |
| Mustelidae | *Ictonyx* |  |  | 1 | 2 | 24.7 | 35 | 43 | 38.5 | 12.4 |
| Mustelidae | *Lutra* |  |  | 4 | 8 | 44.3 | 32 | 39 | 74.5 | 22.1 |
| Mustelidae | *Martes* |  |  | 5 | 11 | 34.3 | 34 | 41 | 52.6 | 17.2 |
| Mustelidae | *Meles* |  |  | 1 | 3 | 39.9 | 29 | 36 | 76.2 | 20.0 |
| Mustelidae | *Mellivora* |  |  | 1 | 2 | 51.0 | 33 | 41 | 75.9 | 25.5 |
| Mustelidae | *Mustela* |  |  | 10 | 16 | 24.2 | 39 | 48 | 33.0 | 12.1 |
| Mustelidae | *Poecilictis* |  |  | 2 | 2 | 21.6 | 38 | 45 | 31.2 | 10.8 |
| Mustelidae | *Taxidea* |  |  | 1 | 4 | 40.8 | 33 | 41 | 71.3 | 20.4 |
| Mustelidae | *Vormella* |  |  | 1 | 1 | 22.1 | 46 | 53 | 39.6 | 11.1 |
| Procyonidae | *Bassaricyon* |  |  | 1 | 1 | 42.2 | 28 | 34 | 50.1 | 21.1 |
| Procyonidae | *Bassaricyon* |  |  | 1 | 1 | 30.4 | 33 | 41 | 55.8 | 15.2 |
| Procyonidae | *Nasua* |  |  | 2 | 4 | 44.3 | 34 | 39 | 78.0 | 22.2 |
| Procyonidae | *Nasuella* |  |  | 1 | 2 | 30.8 | 24 | 29 | 57.2 | 15.4 |
| Procyonidae | *Potos* |  |  | 1 | 4 | 27.1 | 37 | 45 | 48.2 | 13.5 |
| Procyonidae | *Cercoleptes* |  |  | 1 | 1 | 32.8 | 36 | 44 | 51.2 | 16.4 |
| Felidae | *Smilodon* | *fatalis* | LACMHC2001-2 | 1 | 2 | 95.8 | 113 | 123 | 164.3 | 47.9 |
| Felidae | *Hoplophoneus* | *primaevus* | AMNH11858 | 1 | 1 | 77.3 | 51 | 59 | 108.4 | 38.7 |
| Felidae | *Sansanosmilus* | *palmidens* | uncatalogued | 1 | 1 | 100.9 | 73 | 77 | 107.6 | 50.4 |
| Felidae | *Dinobastis* | *serus* | TMM-933-3582 | 1 | 1 | 86.0 | 54 | 58 | 163.7 | 43.0 |
| Felidae | *Homotherium* | *crenatidens* | CB-06 | 1 | 1 | 105.9 | 67 | 75 | 188.0 | 53.0 |
| Felidae | *Megantereon* | *cf.nihowanensis* | BC-120 | 1 | 1 | 78.3 | 82 | 87 | 142.7 | 39.1 |
| Felidae | *Metailurus* | *major* | PMU_M-3841 | 1 | 1 | 65.5 | 52 | 59 | 134.9 | 32.7 |
| Felidae | *Metailurus* | *minor* | PMU_M-3837 | 1 | 1 | 41.1 | 46 | 50 | 99.1 | 20.5 |
| Felidae | *Xenosmilus* | *hodsonae* | BIOPSI-101 | 1 | 1 | 107.3 | 60 | 72 | 167.0 | 53.6 |
| Felidae | *Amphimachairodus* | *giganteus* | MNCN_CAST | 1 | 1 | 110.9 | 61 | 57 | 223.1 | 55.4 |
| Nimravidae | *Dinictis* | *squalidens* | AMNH8777 | 1 | 1 | 74.3 | 77 | 83 | 103.5 | 37.2 |
| Nimravidae | *Dinictis* | *felina* | BC-603 | 1 | 1 | 64.0 | 57 | 64 | 119.9 | 32.0 |
| Nimravidae | *Nimravus* | *gomphodus* | AMNH6933 | 1 | 1 | 66.3 | 47 | 52 | 141.5 | 33.1 |
| Nimravidae | *Hoplophoneus* | *occidentalis* | CB-18 | 1 | 1 | 130.6 | 77 | 85 | 164.0 | 65.3 |
| Nimravidae | *Hoplophoneus* | *sicarius* | CB-07b | 1 | 1 | 101.9 | 75 | 79 | 127.8 | 50.9 |
